# Physiological relevance of epithelial geometry: New insights into the standing gradient model and the role of LI cadherin

**DOI:** 10.1371/journal.pone.0208791

**Published:** 2018-12-21

**Authors:** Yana Vereshchaga, Nikita Arnold, Werner Baumgartner

**Affiliations:** 1 Institute of Biomedical Mechatronics, Johannes Kepler University Linz, Linz, Austria; 2 Institute of Experimental Physics/Soft Matter Physics, Johannes Kepler University Linz, Linz, Austria; University of Colorado Boulder, UNITED STATES

## Abstract

We introduce a mathematical model of an absorbing leaky epithelium to reconsider the problem formulated by Diamond and Bossert in 1967: whether “… some distinctive physiological properties of epithelia might arise as geometrical consequences of epithelial ultrastructure”. A standing gradient model of the intercellular cleft (IC) is presented that includes tight junctions (TJ) and ion channels uniformly distributed along the whole cleft. This nonlinear system has an intrinsic homogeneous concentration and the spatial scale necessary to establish it along the cleft. These parameters have not been elucidated so far. We further provide non-perturbative analytical approximations for a broad range of parameters. We found that narrowing of the IC increases ion concentration dramatically and can therefore prevent outflow through tight junctions (TJs) and the lateral membrane, as long as extremely high luminal osmolarities are not reached. Our model predicts that the system is to some extent self-regulating and thereby prevents fluxes into the lumen. Recent experimental evidence has shown that liver-intestine (LI) cadherin can control the up/down flux in intestines via regulation of the cleft width. This finding is in full agreement with predictions of our model. We suggest that LI-cadherin may increase water transport through epithelia via sequential narrowing of the cleft, starting from the highest concentration area at the beginning of the cleft and triggering a propagating squeezing motion.

## Introduction

Epithelia cover the inner and outer surfaces of most animals, and thus form the primary barrier across which transport into and out of the body occurs. Several epithelia absorb or secrete specific fluids, such as bile, gastric juice, urine, sweat and cerebral fluid. The transported fluid may be hypotonic, isotonic or hypertonic compared to blood plasma. About a century ago [1], it was found that a number of epithelia are capable of absorbing fluid (i.e. water and dissolved ions) in the absence of an electrochemical driving force (isotonic transport). The original work of Curran and Solomon [2] reported that NaCl is absorbed in a rat small intestine in the absence of an electrochemical gradient between lumen and interstitial tissues. Similar behavior was found for the renal proximal tubule of the amphibian Necturus maculosa [3], and for rabbit gall bladder [4]. In vitro preparation of rat intestine was found that water flows from mucosa to serosa even though the osmolarity in mucosal solution is greater than in serosal one [5] which is uphill transport from lumen to interstitial tissues. Such a case is observed in the intestine of teleost fishes—water absorption happens against osmotic gradient [6,7]. That is how they maintain water balance by drinking the hyperosmotic sea water and excreting salt through the gills afterwards. This enigmatic property of epithelia triggered the mathematical community to develop models explaining the phenomenon.

**Fig 1:**
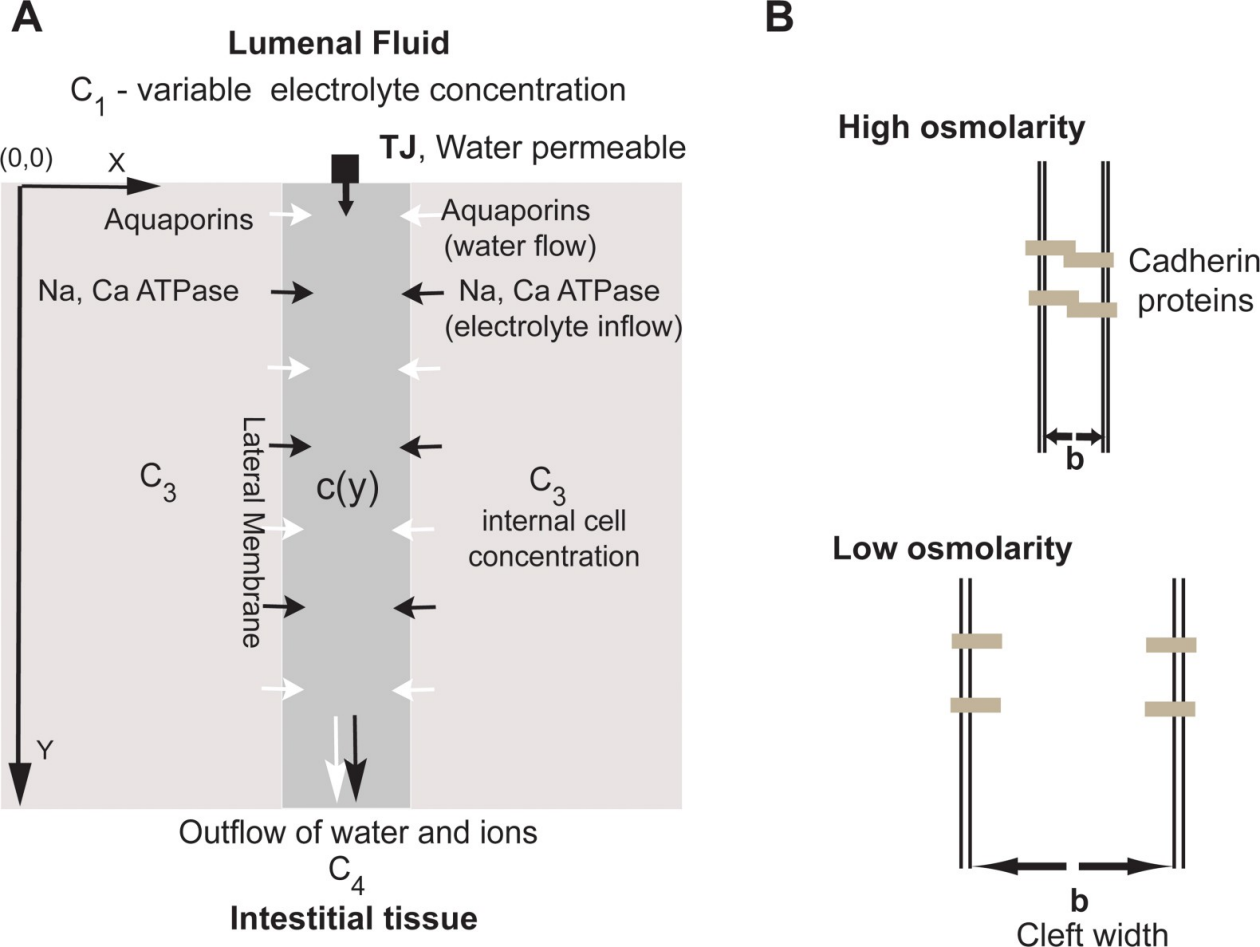
Model of water and electrolyte transport through simple epithelia and IC width regulation via LI-cadherins. a) The model comprises four compartments, which are (1) the lumen of the organ (e.g. the gut), (2) the lateral intercellular cleft (IC), where the values of *c*(*y*), *v*(*y*), *p*(*y*) are calculated, (3) the cytoplasm of the cell, and (4) the interstitial tissue. In the lumen a given concentration of electrolytes is assumed. The tight junctions (TJ) separate the lumen (1) and the IC (2), and are assumed to be impermeable to the electrolyte and permeable to water with a permeability coefficient *k*_*TJ*_. The concentration of electrolytes in the cytoplasm is assumed to be constant *c*_3_ (except in the case in which we explored its influence on the direction of water flow). ATPases are assumed to pump the electrolyte through the lateral membrane into the lateral intercellular cleft. The interstitial tissue is assumed to exhibit a constant electrolyte concentration *c*_4_, which is maintained by the blood vessels located there. The important compartment is the IC. Water enters this compartment through the TJ, aquaporins or from the interstitial tissue. Ions enter through the lateral membrane due to the ATPases and leave the IC due to diffusion and due to the water flux, flushing the lateral intercellular cleft. b) The width of the lateral intercellular cleft b depends on the binding activity of the 7D-cadherins, which in turn depends on the extracellular Ca^2+^ level. High Ca^2+^ concentration triggers LI-cadherin binding and low one courses protein disruption. In the publication [12] we reported the results with a designed peptide that disrupts the LI-cadherins in the epithelium CACO_2_ and the cleft becomes broader as a result.

Several models suggest how epithelia accomplish an “isotonic/uphill” water flux in rat proximal tubules [8], toad intestine [9], and rat intestine [10]. The classical model called *standing gradient theory*, introduced by Diamond-Bossert [11], explains an isotonic transport (i.e. the concentration in the interstitial tissues equals the concentration in the cytosol, *c*_3_ = *c*_4_ refer to Fig 1). It states that ions transported through the intercellular cleft (IC) lateral membrane create a hypertonic region in the paracellular space that serves as the local osmotic driving force, which allows water to pass the transepithelial layer in the absence of electrochemical gradient between lumen and interstitial tissues. Numerical experiments showed that the water flux depends on water permeability of water pores in the lateral membrane, ion transport rate and geometry of the epithelium.

Segel provided perturbative analytical solutions of the governing equations [13]. Both fundamental works [11,13] considered the presence of ion channels only in the first 10%-30% of IC length, near the channel apex. Such a choice yields the predicted osmolarity of an emergent fluid close to the osmolarity in interstitial tissues, which enhances the equilibration between them. At the same time, available experimental studies suggest a uniform distribution of solute pumps along IC [14–17]. This fact was reflected in an analytical model for “uphill” water transport (the condition then *c*_3_>*c*_4_, see Fig 1) suggested by Weinstein and Stephenson [18]. Reported analytical approximations [13,18–20] employ perturbation procedure considering first 1–2 terms of the expansion to obtain *linear* models for different ranges of dimensionless parameters.

Since a mathematical problem possesses a strong non-linearity, perturbative approaches fail for many combinations of parameters relevant in real experiments. The full nonlinear model can be solved numerically, but this often does not uncover important trends, or elucidate the influence of all factors. To circumvent these deficiencies, we discuss the important scales of the full nonlinear model, and rigorously define the concept of a “long” and “short” cleft. For “long” clefts, we derive *exact* analytical solutions of a full *nonlinear* problem, which are valid “almost everywhere”. Despite strong diffusion, constant ion concentration is always established near the entrance part of such clefts. For “short” clefts, we develop *approximate* analytical solutions, based on global conservation laws. They have wider applicability than those based on perturbative linearization. All results are compared with the numerical solutions of the full nonlinear problem. We show, that strong changes in ion concentrations and water velocity along the IC may occur not due to uneven distribution of solute pumps, but only due to interplay between the diffusion, convection and boundary conditions. In addition to water flow through lateral membrane and open end of the IC, our model can also account for tight-junction (TJ) pores in leaky epithelia. The theoretical predictions are compared with the recently published experimental results [12] which suggest the regulation of paracellular transport via liver-intestine cadherins (see below). This idea with a combination of basic modeling was offered earlier in 2011 [10]. The current model is much more advantageous, since it deals with a full nonlinear convection-diffusion problem and incorporates aquaporins influence in the lateral membrane of the IC.

Various adhesion receptors exist in the membranes of intercellular cleft (IC). Some of them regulate the water and ion fluxes through tight junctions (TJ). TJs seal the lateral cleft [21–23] and are localized mostly lumenally. Thin bands of plasma-membrane proteins in TJs control the transport between lumen and epithelium as well as between enterocytes. Epithelia can be classified as “leaky” or “tight” in respect to lumen with water pores 40 Å and 4 Å in diameter accordingly [24]. Mammals have leaky epithelium in the small intestine.

Some epithelia have additional adhesion receptors which are uniformly distributed along the whole lateral membrane [25–27] rather than lumenally: 7D-cadherins can be found particularly in simple epithelia that transport liquid, for instance, in the kidney, the liver and the small intestine [28]. They are most prevalent in the small intestine in the form of LI-cadherin (liver-intestine cadherin). LI-cadherin binding responds to small changes in the extracellular Ca^2+^ concentration below the physiological plasma concentration with a high degree of cooperativity (Fig 1B). It has therefore been suggested that LI-cadherin might regulate the IC width [10,29–31]. We have shown recently [12] for the first time that disruption of LI-cadherin binding, which results in widening of the IC, can alter the water flux direction from positive (from lumen to interstitial tissue) to negative (into the lumen) under hypertonic conditions. Considering hyper- and hypoosmotic conditions in the lumen, we compared the experimental results [12] with our theory. The findings suggest, that LI-cadherins should significantly contribute to a regulation of water absorption in response to a lumen osmolarity switch.

Our theoretical analysis has the following goals. a) describe the stationary distribution of the ion concentration and velocity along the cleft; b) find intrinsic (dimensionless) parameters, which define the water flow in the cleft; c) compare the theoretical predictions with experimental results [12] and suggest potential mechanisms that cells use to regulate water absorption from the lumen under hyper-osmotic conditions.

## Results

### Theoretical model description: Concentration and velocity profiles

The cross-section of a paracellular channel (side) is approximated by a long, narrow rectangle with semipermeable walls for ions and water. We assume that at the closed end (*y* = 0) a TJ pore is able to conduct water; at the open end (*y* = *L*), electrolyte and water move freely into and out of the cleft (Fig 1). The system parameters are listed in Table 1.

**Table 1:** List of constants.

Constants	Value	Units
Water permeability [32]	*f*_*aq*_ = 5⋅10^−14^	*cm*⋅*s*^−1^
Aquaporin density^a^ [33,34]	*n*_*aq*_ = 10^3^⋅10^8^	*cm*^−2^
Ion flux through lateral membrane [4,35]	*J*_ions_ = 18.5⋅10^−9^	*mol*⋅*cm*^−2^⋅*s*^−1^
Diffusion coefficient	*D* = 1⋅10^−5^	*cm*^2^⋅*s*^−1^
Intercellular Cleft width	*b* = 40÷400	*nm*
Intercellular Cleft length	*L* = 20÷100	*Μm*
Viscosity	*μ* = 0.7⋅10^−2^	*g*⋅*cm*^−1^⋅*s*^−1^
Temperature, Gas constant	*RT* = 310.15⋅8.314⋅10^7^	*g*⋅*cm*^2^⋅*s*^−2^
Molar volume of water	*V*_*wat*_ = 18	*cm*^3^⋅*mol*^−1^
TJ pore width^b^	*TJ*_*pore*_*width*_ = 2⋅10^−9^	*m*
TJ hydraulic permeability [24]^c^	*P*_*f*_ = 13.1⋅10^−3^	*cm*⋅*s*^−1^
Osmolarity in Lumen	*c*_1_ = 200÷1000	*mM*
Osmolarity in the enterocyte/interstitial tissues	*c*_3_,*c*_4_ = 300^d^	*mM*

^a^The order of values differs from [11] for aquaporin permeability ($1\times 10^{-6}÷1\times 10^{-4}\left[\frac{cm}{s}\right]$)

^b^This value was chosen referring to a moderately leaky epithelium, considering that leaky and tight epithelia have pore widths of 4*nm* and 0.4*nm*, respectively. [24]

^c^This value for leaky epithelium was taken from [24]. The hydraulic permeability of TJ has been assessed for different epithelia in publications [9,23,36]; the values vary. The velocity at *Y* = 0 in Eq (5) for a moderately leaky epithelium was calculated from the permeability of the TJ pore using $k_{TJ}=P_{f}\frac{V_{wat}TJ_{pore\_width}}{RTb}$.

^d^This parameter was changed only for the case in which we explored a possible changes in osmolarity inside the cell.

The system has two essential dimensions–along the cleft (*y*-axis) and along the *x*-axis due to water emerging from aquaporin pores. After averaging the velocity and concentration along the *x*-direction (see explanations in the S1 File) the time-independent problem can be described in a simplified way (below). Main variables are the total ion concentration *c* = *c*(*y*) and velocity of the fluid in the channel *v* = *v*(*y*) at position *y*. The velocity is considered positive in positive *y*-direction.

The transport of ions by convective flow of water and diffusion obeys a convection-diffusion equation:

$$-D\frac{\partial^{2}c}{\partial y^{2}}+\frac{\partial (vc)}{\partial y}=\frac{2j}{b},$$ (1)

Here the source function 2*j/b* at the right hand side accounts for ion flux through lateral walls. *b* is the cleft width, *j* is the ion flux through the lateral membrane due to Na^+^, Ca^2+^ and ATPase, and *D* is the ion diffusion coefficient.

The velocity of the water emerging from the lateral membrane via aquaporin pores depends on the ion concentration along the cleft:

$$v_{0}(y)=f_{aq}(c-c_{3}),$$ (2)

where *c*_3_ is the concentration inside the enterocyte. The aquaporin conductivity is defined as

$$f_{aq}=k_{aq}V_{wat}n_{aq},$$ (3)

where *n*_*aq*_ and *k*_*aq*_ are equally distributed [37] aquaporin density and permeability in the lateral membrane, and *V*_*wat*_ is the molar volume of water (Table 1).

Velocity of water *v*(*y*) builds up along the cleft as a result of water influx from aquaporins at both lateral membranes. Due to mass (and volume) conservation, this can be expressed as:

$$\frac{\partial v}{\partial y}=\frac{2v_{0}}{b}=f_{aq}\frac{\partial c}{\partial y}.$$ (4)

The difference in osmotic pressures defines the water flux through the TJ:

$$v_{TJ}=k_{TJ}RT(c(0)-c_{1}),$$ (5)

where *k*_*TJ*_ is the permeability of TJ pores (see footnote “c” to Table 1).

The governing differential Eqs 1 and 4 are to be solved with the following three boundary conditions for the beginning and the end of the duct and along the duct’s walls:

$$\begin{array}{l} (a)\;(-D\frac{\partial c}{\partial y}+vc)[y=0]=0\;;\\ (b)\;c(y=L)=c_{4};\\ (c)\;v(y=0)=v_{TJ}=k_{TJ}RT(c(0)-c_{1}); \end{array}$$ (6)

Condition in Eq 6A reflects the absence of (axial) ion flux at *y* = 0, which is the sum of diffusion and convection fluxes. Eq 6B indicates that concentration at the open end of the cleft equals the electrolyte concentration of interstitial tissue (Fig 1). Eq 6C describes velocity through TJ–it is defined by the difference between the lumen concentration *c*_1_ and concentration in the beginning of the cleft *c*(0) (Fig 1).

Using condition (a) from Eq 6, we can integrate the concentration Eq 1 once, since *b*, *j* and *D* are constants. If *j* changes along *y*, the integration is still possible, but the subsequent analysis becomes different. Hydrostatic pressure contributions to the aquaporin and TJ conductance are neglected, since they are small. Typical values are in the range Δ*c* = 100*mM*, and therefore *RT*Δ*c* = 2.58⋅10^6^ CGS, while Δ*p* = 0.1*atm* = 10^5^ CGS, which is about 26 times smaller. Under these assumptions, the system of Eq 6 is reduced to the following nonlinear and non-autonomous (with respect to the spatial variable *y*) system of two first order Eq *7A and 7B* with boundary conditions *7c*,*d*.

$$\begin{array}{l} (a)\;-D\frac{\partial c}{\partial y}+vc=\frac{2jy}{b};\\ (b)\;\frac{\partial v}{\partial y}=\frac{2f_{aq}}{b}(c-c_{3});\\ (c)\;c(L)=c_{4};\\ (d)\;v(0)=k_{TJ}RT(c(0)-c_{1}). \end{array}$$ (7)

The interplay between various physiological constants in the Eq 7 define water flux direction. To analyze this system analytically, we first discuss intrinsic properties of the concentration and velocity profiles.

### Inherent constant concentration far from the boundaries

With various parameters and boundary conditions, the Eq 7 produce very dissimilar solutions, describing physiologically different regimes. However, this *nonlinear* problem (without boundary conditions) also has a simple solution with constant concentration and linear velocity profile. This is important, because such a behavior sets in “far” from the boundaries, and thus develops in long clefts. We define “long” more rigorously later. To find this solution, we write the concentration and velocity in the form:

$$\begin{array}{l} (a)\;c=c_{hom}+\delta c\\ (b)\;v=v_{hom}(y)+\delta \nu , \end{array}$$ (8)

Here, *c*_*hom*_ = *const* is spatially homogeneous concentration, and *v*_*hom*_(*y*) is the (yet unknown) stationary velocity profile. Deviations from these values are *δc* and *δv*, they become important near the edges of the cleft (see subsequent sections) and are disregarded for now.

For constant concentration *c* = *c*_*hom*_ the diffusive term in Eq 7A disappears. The remaining terms produce a linear velocity profile *v*_*hom*_(*y*) = *Ay* Together with Eq 7B this yields:

$$\begin{array}{l} c_{hom}^{2}-c_{3}c_{hom}-j/f_{aq}=0\;\Rightarrow \\ c_{hom}=\frac{c_{3}}{2}\left(1+\sqrt{1+\frac{4j}{f_{aq}c_{3}^{2}}}\right)>c_{3},\;A=\frac{2j}{bc_{hom}} \end{array}$$ (9)

The resulting concentration *c*_*hom*_ is related to, but larger than *c*_3_. It is further influenced only by the lateral ion flux *j* and aquaporins conductivity *f*_*aq*_. Linear velocity profile defines a natural velocity scale at the cleft exit. It is influenced by the cleft length *L* and width *b*:

$$v_{hom}(y)=Ay\;\Rightarrow \;v_{hom}(L)=AL=\frac{2jL}{bc_{hom}}$$ (10)

In this regime, the constant osmotic water influx through aquaporins linearly increases the velocity along the cleft due to volume conservation (incompressibility). With homogeneous influx of ions *j* = *const*(*y*), their overall transport also increases linearly. It happens via advection only, without diffusion (Eq 7A). This behavior dominates in long clefts. Near the edges, these profiles are modified by the boundary conditions, especially near *y* = *L*, where *c*(*L*) = *c*_4_ ≠ *c*_*hom*_.

**Fig 2:**
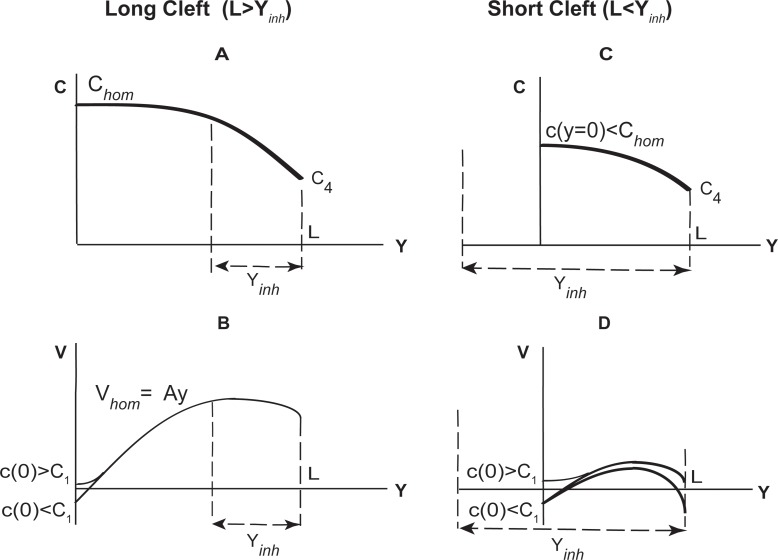
**Schematical graphs of the ion concentration *c* and water velocity *v* in long (*L*>*y***_***inh***_**) (A-B) and short (*L*≤*y***_***inh***_**) (C-D) clefts.** Physiologically related scenarious are shown. Non-physiological ones, for example, when *c*(*L*) is much higher than spatially homogeneous concentration *c*_*hom*_, are not shown here.

**Fig 3:**
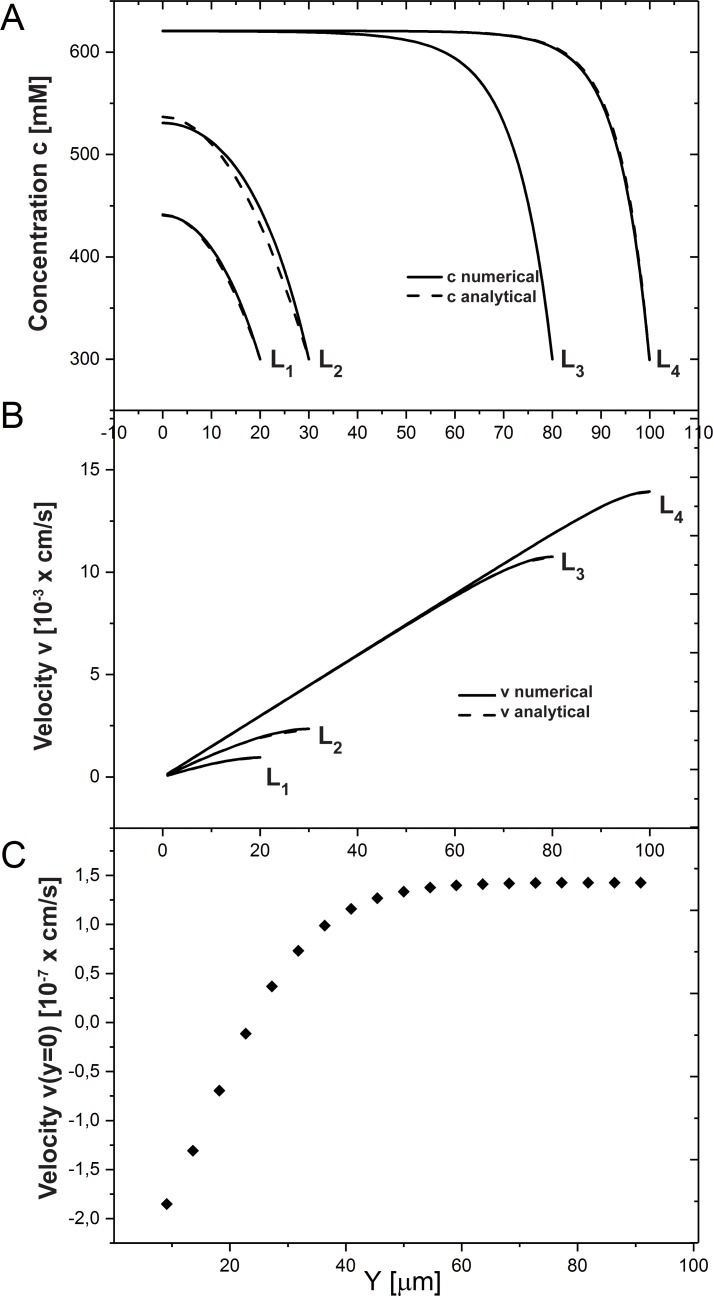
Influence of cleft length on ion concentration (c) and water velocity (v). Concentration (A) and velocity (B) profiles (analytical approximation and numerical solutions (see S1 File for details)) in the case of long (***L*>*y***_***inh***_,*L*_3_ = 80 *μm*,*L*_4_ = 100 *μm*) and short (***L*≤*y***_***inh***_, *L*_1_ = 20 *μm*, *L*_2_ = 30 *μm*) clefts are shown. (C) Change in velocity at the cleft TJ end. Increase in cleft length reverses the water flux in the TJ from negative to positive. The following system parameters were used: *C*_3_ = 290 *mM*, *C*_4_ = 300 *mM*, *C*_1_ = 600 *mM*.

Concentration and velocity profiles demonstrate qualitatively different behavior for different cleft lengths (Figs 2 and 3), which requires further analysis. Below we introduce the concept of “long” and “short” clefts, and provide an analytical approximations for both cases (Eqs 13 and 15).

### Analytical approximations for concentration and velocity; spatial scale to establish a homogeneous regime

Let us now study the deviations from the homogeneous solution Eqs 9 and 10. They become essential near the boundaries and in shorter clefts. We substitute expressions Eq 8 into the equations Eq 7, cancel zero-order terms with *c*_*hom*_,*v*_*hom*_(*y*) = *Ay*, and retain all other terms, including the nonlinear product *δcδv*. This results in:

$$\begin{array}{l} -D\delta c_{y}+\delta cAy+c_{hom}\delta v+\delta c\delta v=0,\;\delta c(L)=c_{4}-c_{hom},\\ \delta \nu_{y}=\frac{2f_{aq}}{b}\delta c,\;\delta \nu (0)=k_{TJ}RT(\underset{c(0)}{c_{hom}+\delta c(0)}-c_{1})\approx 0, \end{array}$$ (11)

In the last expression we assumed that the velocity at the cleft entrance is small compared to the characteristic velocity at its end (Eq 10).

For typical values we obtain:

$$\frac{\delta \nu (0)}{v_{hom}(L)}=\frac{k_{TJ}RTc_{1,3,4,hom}}{\underset{v_{hom}(L)}{2jL/bc_{hom}}}∼\frac{k_{TJ}RTbc^{2}}{2jL}∼2.14\times 10^{-5}$$ (12)

The analysis of Eq 11 (see S1 File) results in the following expressions for long and short clefts.

For long clefts with $L\geq y_{inh}=\sqrt{\frac{Dbc_{hom}}{j}}$, the differential Eq 11 have approximate solutions Eqs 25(b,c) in S1 File. In dimensional variables they become:

$$\begin{array}{l} v=\frac{2jy}{bc_{hom}}+\frac{Df_{aq}c_{hom}(c_{4}-c_{hom})}{jy}e^{-\frac{j}{c_{hom}bD}(y^{2}-L^{2})}{\left(\frac{y}{L}\right)}^{\frac{f_{aq}c_{hom}^{2}}{j}}\\ c\approx c_{hom}+(c_{4}-c_{hom})e^{-\frac{j}{c_{hom}bD}(y^{2}-L^{2})}{\left(\frac{y}{L}\right)}^{\frac{f_{aq}c_{hom}^{2}}{j}},\;c_{0}\approx c_{hom} \end{array}$$ (13)

The analytical approximations Eq 13, together with numerical solutions (see S1 File), are shown in Fig 3 for cleft length 20,30,80, and 100 *μm*. The exponents in formulas Eq 13 contain a *characteristic spatial inhomogeneity scale*:

$$y_{inh}=\sqrt{\frac{Dbc_{hom}}{j}}=\sqrt{\frac{Dbc_{3}\left(1+\sqrt{1+\frac{4j}{f_{aq}c_{3}^{2}}}\right)}{2j}}\approx \underset{b=40nm}{12.2}\;\mathrm{to}\;\underset{b=400nm}{38.5}\;\mu m$$ (14)

**Fig 4:**
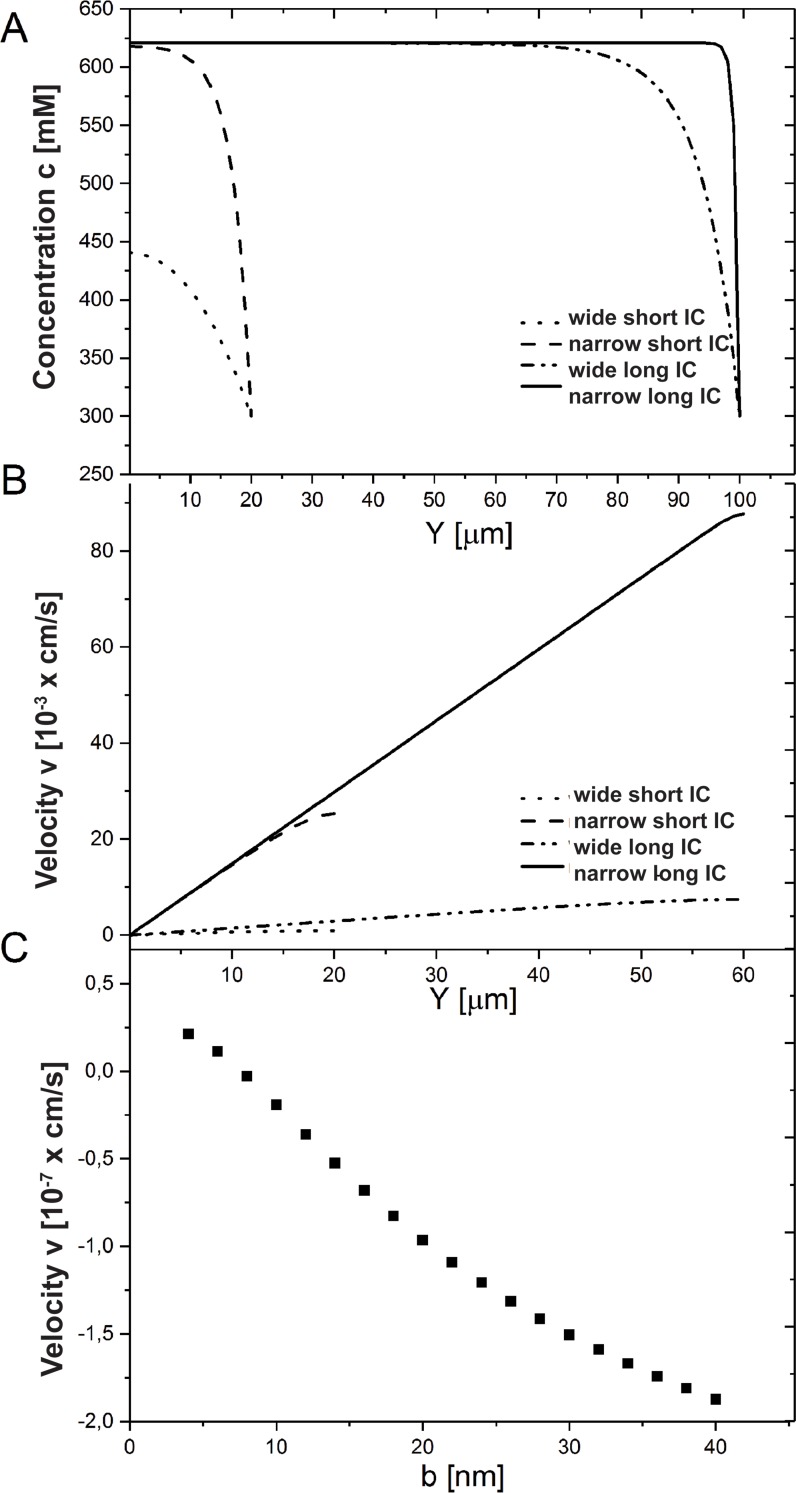
Influence of cleft width on ion concentration and water velocity. (A) The concentration increases as the cleft width gets decreased from 400 nm (wide IC) to 40 nm (narrow IC). The results for a short *L* = 20 *μm*≤*y*_*inh*_ and long *L* = 100 *μm*>*y*_*inh*_ cleft are shown. (B) The velocity growth is shown for short and long clefts (*L* = 60 *μm*>*y*_*inh*_) as IC width decreases. (C) The flux direction (velocity *v*(0)) through the TJ alters from negative to positive one as IC becomes wider. The following system parameters were used: *C*_3_ = 290 *mM*, *C*_4_ = 300 *mM*.

Here, *c*_*hom*_ is calculated with values from Table 1 for narrow and wide clefts (*b* = 40 *nm* and 400 *nm*). For long clefts with *L*≫*y*_*inh*_, boundary disturbance introduced by the concentration *c*_4_ ≠ *c*_*hom*_ at the cleft outlet *y* = *L* relaxes over a distance ~*y*_*inh*_, and homogeneous solution Eqs 9 and 10 establishes in the region 0<*y*<*L*−*y*_*inh*_. Since $y_{inh}\propto \sqrt{b}$, this happens easier in narrow clefts (see Discussion and Fig 4). For clefts shorter than *y*_*inh*_, homogeneous solution Eqs 9 and 10 cannot develop.

For short clefts with $L\ll \frac{y_{inh}}{\sqrt{2(1+\frac{f_{aq}c_{hom}^{2}}{j})}}$, the global conservation laws for Eq 11 produce parabolic approximations Eq 27 (discussed in section 2 of the S1 File). In dimensional form they read:

$$\begin{array}{l} c\overset{\left(1\right)}{\approx }c_{hom}+c_{hom}(C_{0}+(\frac{c_{4}-c_{hom}}{c_{hom}})\frac{y^{2}}{L^{2}})\overset{\left(2\right)}{\approx }c_{hom}+(c_{4}-c_{hom})\left(1+(1+\frac{f_{aq}c_{hom}c_{4}}{j})(\frac{y^{2}-L^{{}^{2}}}{y_{inh}^{2}})\right)\\ v\overset{\left(1\right)}{\approx }\frac{2jy}{bc_{hom}}+D(C_{0}+(\frac{c_{4}-c_{hom}}{c_{hom}}-C_{0})\frac{y^{2}}{3L^{{}^{2}}})\overset{\left(2\right)}{\approx }\frac{2jy}{bc_{hom}}+\frac{c_{4}-c_{hom}}{c_{hom}}Dy\left(1+(1+\frac{f_{aq}c_{hom}c_{4}}{j})(\frac{\frac{1}{3}y^{2}-L^{{}^{2}}}{y_{inh}^{2}})\right) \end{array}$$ (15)

The first of approximations from Eq 15 (marked with (1) above the equality signs) are shown in Fig 3 for cleft length of 20 and 30 *μm*. They are much more accurate than perturbative results [20] for such clefts. The value *C*_0_ there should be taken from the cumbersome 1^st^ of Eqs 29 in S1 File. The second equalities in Eq 15 (marked with (2) there) better illustrate the influence of the main parameters, but they are valid only for very short clefts (≈10 *μm*). For example, they contain inhomogeneity length *y*_*inh*_, albeit slightly modified by the *c*_4_ value. The boundary values *c*(0) and *v*(*L*) follow from expressions Eq 15 applying *y* = 0,*L*.

We do not address here the question how the concentration of the emergent fluid (efflux concentration *c*_*e*_ Eq 18–21 (in S1 File) in ref. [38], in our case equal to $c_{e}=\frac{2jL}{bv(L)}$) equilibrates outside of the cleft with the concentration within interstitial tissues. We assume that this equilibration happens via 3D convection-diffusion in the vicinity of the cleft outlet, on the spatial scale related to geometry of IC cross-section–few of its effective diameters, or lengths of its largest edge. But we see, that as in the work of Diamond and Bossert [11] narrowing of the cleft decreases *c*_*e*_. In our case *c*_*e*_ stays in the range 600-700mM as cleft narrows.

Below we demonstrate how changes in the intrinsic parameters can alter water flux through TJs and lateral membrane, resulting in the overall outflow through the open end of IC.

### Water flux is defined by cleft geometry

Two of the main parameters that govern system behavior are spatially homogeneous concentration $c_{hom}=\frac{c_{3}}{2}\left(1+\sqrt{1+\frac{4j}{f_{aq}c_{3}^{2}}}\right)$ and spatial inhomegeneity scale $y_{inh}=\sqrt{\frac{Dbc_{hom}}{j}}$. They depend on ATPases (*j*), aquaporins (*f*_*aq*_), internal cell concentration (*c*_3_) and clefts width (*b*). The enterocyte can regulate all these parameters to modify water transport (see the influence of ATPases and aquaporins expression level (S1 Fig) on the concentration along the cleft in S1 File). The change of these values occurs on different time scales.

### Water flux through tight junctions depends from cleft length

The water flux direction through the TJ complex is defined primarily by the difference in osmotic pressure in lumen (*c*_1_) and paracellular cleft due to Eq 6C. Therefore if *c*(0)>*c*_1_, the flux is positive (towards the interstitial tissue), and vice versa. Therefore concentration does not have a boundary condition at the cleft entrance *y* = 0.

Concentration at the beginning of the cleft *c*(0) depends on the cleft length, as shown in the Fig 3C. Long clefts with *L*>*y*_*inh*_ provide water absorption from lumen despite hyperosmolarity conditions, since in long clefts the concentration at the cleft entrance equals the established spatially homogeneous concentration, *c*(0)≈*c*_*hom*_ (Fig 3A, curves L3, L4). As long as homogeneous concentration *c*_*hom*_ is bigger than lumen osmolarity, the absorption via TJs occurs. For instance, in case of IC length 100 *μm* (Fig 3A), the concentration at the cleft entrance is $c(0)=c_{hom}=\frac{c_{3}}{2}\left(1+\sqrt{1+\frac{4j}{f_{aq}c_{3}^{2}}}\right)\approx 620\;mM$, and it exists over the region 0<*y*<*L*−*y*_*inh*_, where $y_{inh}=\sqrt{\frac{Dbc_{hom}}{j}}\approx 38.5\;\mu m$ for *b* = 400 *nm*.

In case of *L*≤*y*_*inh*_, the system does not fully develop a homogeneous concentration and a linear velocity profile. As a result, the prescribed concentration at the cleft outlet *c*(*L*) = *c*_4_ ≠ *c*_*hom*_ shifts inlet concentration *c*(0) towards *c*_4_ (Fig 3A, curves L1, L2). This shift can be approximated using the first expression in Eq 15 at *y* = 0:

$$c(0)-c_{hom}\approx (c_{4}-c_{hom})\left(1-(\underset{y_{inh}^{-2}}{\frac{j}{Dbc_{hom}}}+\frac{f_{aq}c_{4}}{Db})L^{{}^{2}})\right)$$ (16)

The effect is stronger for shorter clefts, where the ratio *L*/*y*_*inh*_ is smaller (cf. L1 and L2 in Fig 3A). For a given cleft length *L*, the *absolute* shift is bigger for wider clefts (larger *b*), since they have larger *y*_*inh*_ (see Eq 14). From another viewpoint, Eq 17 in S1 File states that inhomogeneity at the outlet *c*_4_−*c*_*hom*_ is *always reduced* at the inlet, but the *relative* reduction is *weaker* for shorter or wider clefts with smaller *L*/*y*_*inh*_ ratio. The dependence on other parameters is more complex, as *c*_*hom*_ in Eq 9 itself depends on them. Hence, cleft geometry, physiological constants and their combination can all strongly modify water fluxes in the IC. We consider several scenarios below.

### Absorption regulation by cleft width

Smaller IC width *b* broadens the homogeneous range 0<*y*<*L*−*y*_*inh*_ since $y_{inh}=\sqrt{\frac{Dbc_{hom}}{j}}$ weakens. For enterocytes with long length *L*>*y*_*inh*_, the cleft width *b* has no effect on the inlet water flow through the TJ, since homogeneous concentration does not depend on *b*. For short clefts with *L*<*y*_*inh*_, width *b* can modify the flux direction through TJ by changing the concentration at the clefts entrance *c*(0) in Eq 7B. Fig 4A shows that with decreasing cleft width the concentration in the cleft entrance increases towards *c*_*hom*_, which is reached when the following condition is satisfied: *L*>*y*_*inh*_. This concentration rearrangement changes the flux direction from negative to the positive (Fig 4C).

As the cleft gets narrower, lateral water flux into IC increases (Fig 4A and 4B). As a result, the overall flux through the open end *v*(*L*)*b* rises tremendously, since the velocity growths faster than IC width decreases (Fig 4C). Therefore absorption rate increases as the cleft gets narrower.

Let us now suggest the mechanism that can avoid dangerously high concentrations in the enterocyte during uphill transport.

### Elevated cell osmolarity can be lowered by water fluxes through lateral membrane

Biologically, osmolarity *c*_3_ inside the enterocyte can rise in response to lumen osmolarity [39]. Thus, it is important to analyze its influence on the system.

Increase in *c*_3_ increases the spatially homogeneous concentration via relation $c_{hom}=\frac{c_{3}}{2}\left(1+\sqrt{1+\frac{4j}{f_{aq}c_{3}^{2}}}\right)>c_{3}$. It facilitates more efficient water absorption through the TJ and therefore provides higher stability of the epithelium to lumen osmolarity variations. It is especially important when the lumen is highly hypertonic. The escalating enterocyte concentration brings the system to an interesting phenomenon: in a proximity from TJs at the cleft segment where *c*(*y*)>*c*_3_ water flows in a positive direction. At the rest of the cleft the velocity is negative since *c*(*y*)<*c*_3_. According to Eqs 2 and 7B, the *lateral* flux changes its direction at the point where *c*(*y*) = *c*_3_ and the *overall* flux changes sign where *v*(*y*) = 0 (which can be found from the Eq 15).

The influence of high internal cell concentrations can be understood in the following way. The potentially dangerously high osmolarity in the cell can be reduced, since the water inflow through the lateral membrane downturns the concentration inside the enterocyte. The gut continues to absorb water through TJ pores. Both mechanisms work in the same direction, in such a self-regulation scenario the system can “kill two birds with one stone”.

### Comparison of simulated absorption with experimental observations

**Fig 5:**
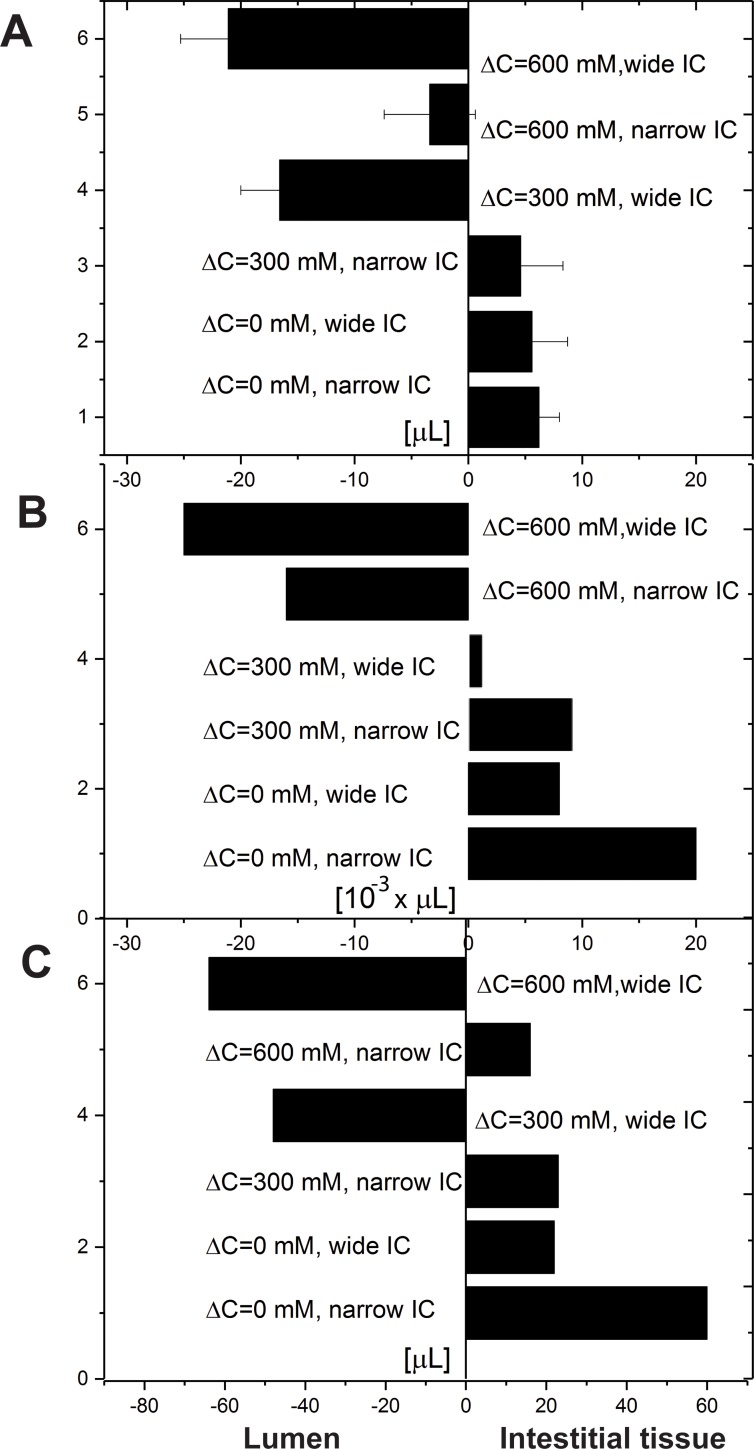
Change in water volume per hour for strong, medium hypertonic, and isotonic osmolarities in the lumen. Osmolarity difference between lumen and interstitial (Δ*c* = *c*_1_−*c*_4_) is accordingly 600, 300, and 0 *mM*. The results are presented for wide (*b* = 400 nm) and narrow (*b* = 40 nm) clefts. (A) Experimentally measured fluxes, as the peptide disrupts LI cadherins binding (the IC widening as shown in Fig 1B with low osmolarity). The figure is adapted from [12]; (B) Water flux change through the TJ, numerical results; (C) Water flux through the open end of the IC as a result of elevated cytosolic concentration *c*_3_. Changes in response to the alteration of lumen osmolarity: 900*mM*, 600*mM* and 300*mM*. The following system parameters were used: *c*_4_ = 300 *mM*,*L* = 20 *μm*.

Recent publication [12] reports experimental data on an alteration of water flux direction as the cleft width changes. The authors designed and introduced a peptide that disrupts the LI-cadherins in the epithelium CACO_2_ (Fig 1B) and the cleft becomes broader as a result. Considering hyper- and isoosmotic conditions similar to the experimental ones (Fig 5A, experimental part of the figure is borrowed from [20]), we compare the system behavior with that predicted by our model.

Fig 5B and 5C shows that the behavior described by our model resembles that reported in [12] (Fig 5A). For strong hyperosmolarity condition narrowing of the cleft results to lower outflow through TJs (Fig 5B) or alter the overall flux direction with increasing of the absorption (Fig 5C).

The amount of water detected experimentally is about 1000 times greater than fluxes through TJ predicted by the model (Fig 5B). The exact pathway was not determined in vitro [12]. Bearing in mind water permeabilities for leaky epithelia suggested by Fischbarg [24] and cell osmolarity around 300 *mM* TJ water flux forms only 0.01%−0.30% of the total water flux at the cleft outlet. Experimentally measured fluxes can be reproduced much better (the qualitative behavior stays the same), via much larger TJ conductivities of the order $(60-100)\cdot 10^{-7}\left[\frac{cm}{s\cdot mmHg}\right]$. Similar values were used in the model for rat kidney proximal tubule [8]. In this circumstance, the TJ water flux would constitute then 10%-100% of the total water flux (at the open duct end), depending on width and lumen osmolarity.

**Fig 6:**
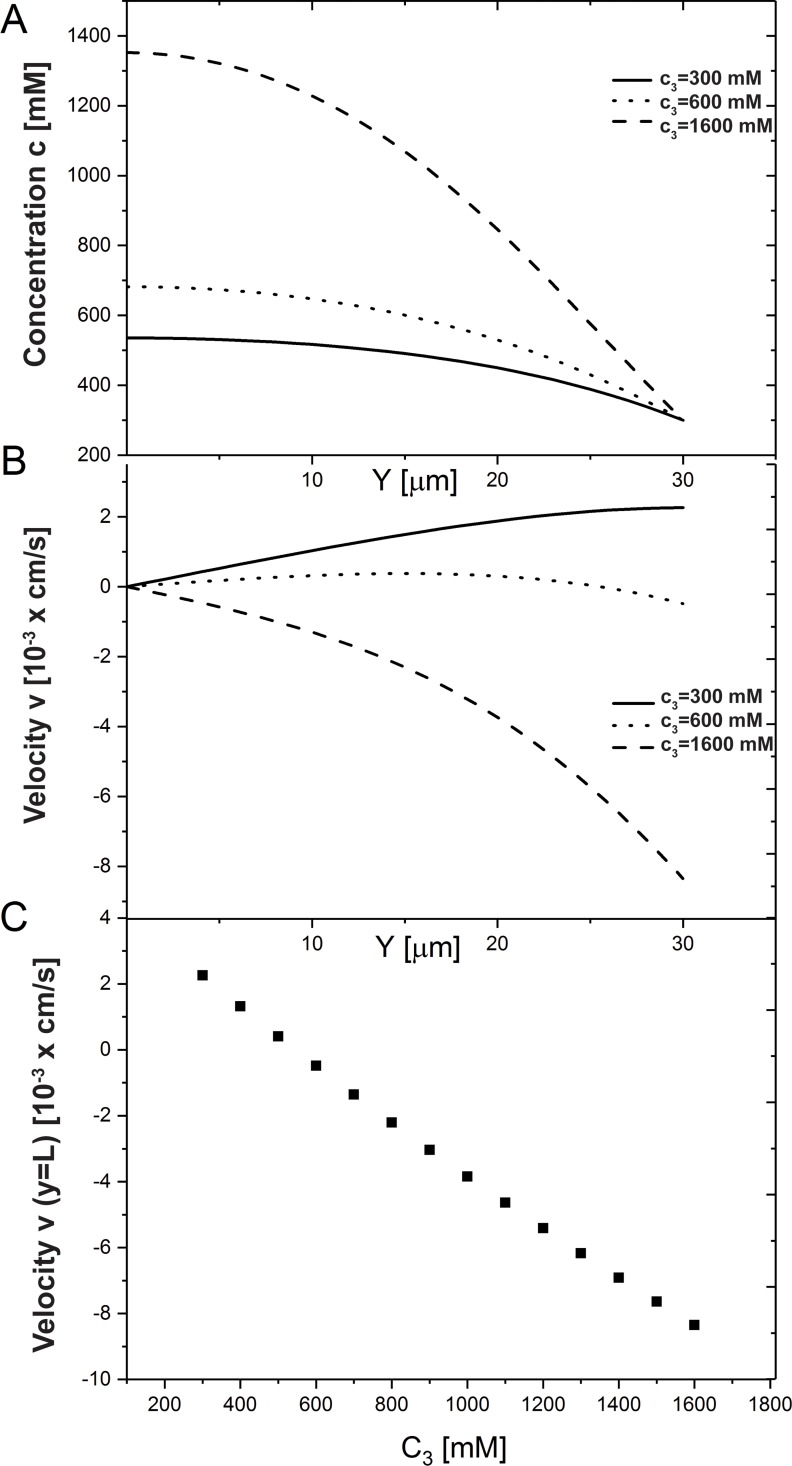
Influence of internal cell concentration *c*_3_. (A) Ion concentration and (B) water velocity profiles along the IC. (C) The velocity at the end of the cleft *v*(*L*) as *c*_3_ increases from 200 to 1600 *mM*. The IC length is *L* = 30*mM*, and *c*_4_ = 300*mM*.

**Fig 7:**
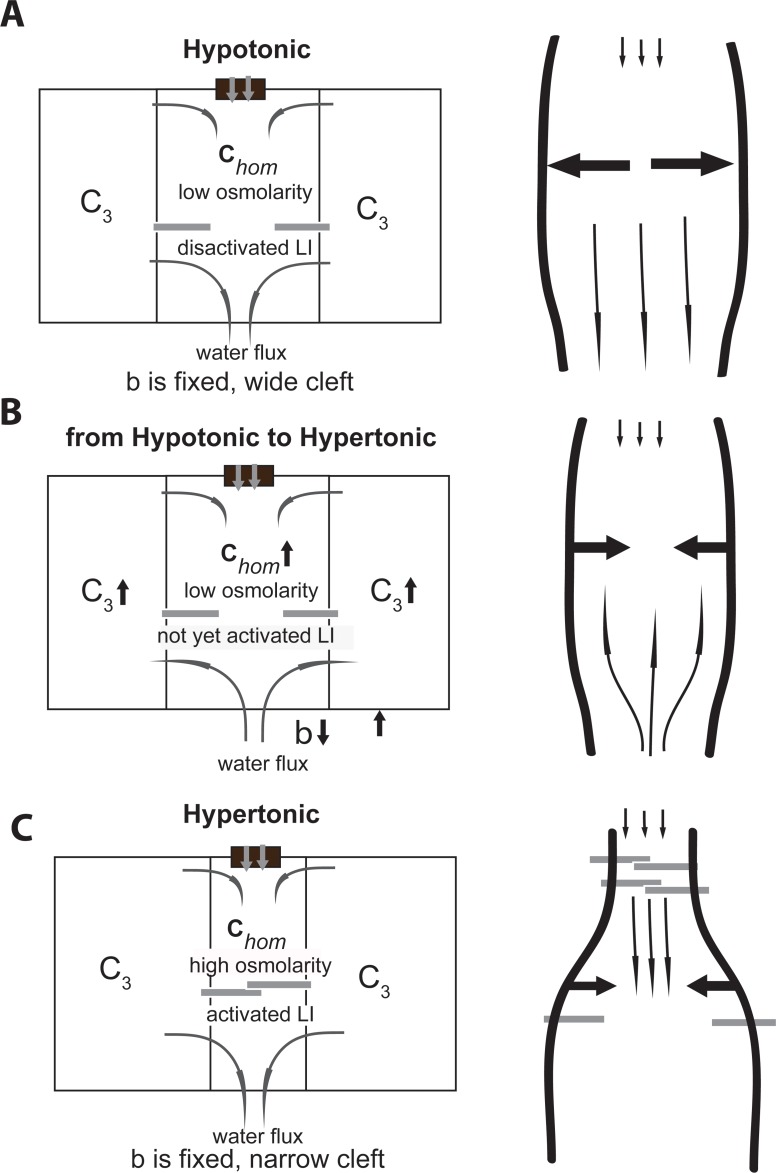
The potential mechanism by which LI-cadherins can squeeze water out of the IC in the course of lumen osmolarity change is introduced schematically. (A) The cleft is wide as LI-cadherins are in deactivated state. (B) Change from hypotomic to hypertonic conditions. LI-cadherins are not yet activated, the internal cell *c*_3_ and the homogeneous concentrations increase. Water flux in the negative direction and through lateral membrane is present. (C) LI-cadherins are activated as the homogeneous concentration *c*_*hom*_ reaches a certain value–LI activation causes the water to be squeezed downwards.

One may question if TJs in the CACO_2_ cell line are sufficiently loose to transport that amount of water. Another scenario to explain the difference in volume scales (Fig 5) is to compare a water flux through lateral membrane (we infer it emerges then in the lumen via apical membrane and we do not model this process here). We assume that lumen and cytosolic osmolarity are linked, and the latter increases as the lumen concentration rises. As a result, water flux into the cell from the cleft emerges (Figs 6 and 7B, S2 Fig). Calculated overall fluxes through IC lateral membrane (or cleft outlet) are qualitatively and quantitatively similar to those found experimentally [12]. For broad clefts, water outflux is observed (Fig 5C). The question remains, whether the negative outflux to the lumen occurs through the TJ in the experiments, or through the lateral membrane.

Despite the fact that for typical parameters (Table 1), the velocities at the beginning of the cleft are extremely low and are in the range of 10^−7^−10^−6^*cm*/*s* (see Eq 12), appreciable water volumes accumulate over hours or days. For example, considering a short epithelium with 30 *μm* height and hypertonic conditions in the lumen with 1000 *mM*, the reverse flux for an area of 1 *m*^2^ is 2 *mL* per day (see S1 File). This means that water flux from the entire intestine amounts to approximately 0.6 *L* (the surface area is generally accepted to be ≈300 *m*^2^). Therefore, regulation of the cleft width in response to lumen osmolarity can be crucial.

## Discussion

Cellular adhesion is of vital importance for the integrity of epithelia to allow for maintaining their selective barrier function. This barrier function consists of a) hindering undesired transport of molecules and b) allow for a physiological selective and directional transport of water, nutrients and other substances.

Adhesion between simple epithelial cells like enterocytes is mainly accomplished by the so called junctional complex (tight junctions, adherens junctions and desmosomes). While the tight junction and the adherens junction are localised close to the apical side at the lateral membrane [21–23], the desmosomes are broadly distributed over the lateral membrane. Besides these cell contacts, so called 7D-Cadherins (LI-cadherin or KSP) are also expressed in some simple epithelia throughout the lateral membrane [28]. It has to be noticed that 7D-cadherins are found in epithelia where water transport under varying osmotic conditions takes place like in the gut, the liver and the kidney. In contrast to classical cadherins, 7D-cadherins have seven extracellular cadherin repeats and a very short cytosolic domain [27]. However, it was shown in the past that 7D-cadherins are functional adhesion molecules. Furthermore it was shown that they exhibit a highly cooperative Ca^2+^-dependence of their binding activity [29] and recently it was found that at least LI-cadherin can influence the water transport through enterocyte-monolayers [12].

Clearly as relatively long adhesion molecules in the lateral membrane 7D-cadherins have the ability to influence the geometry of the lateral intercellular cleft (IC). Hence we tried to understand how the geometry of the IC in simple epithelia influences the transport of ions and most important the transport of water through the epithelial barrier.

For that reason, we developed a standing gradient model of the intercellular cleft including tight junctions and ion channels distributed along the whole cleft uniformly. This allows for the calculation of the osmolarity (ion concentrations) along the IC and thus the local water fluxed driven by these local osmotic gradients. The rather complex nonlinear system describing the ion concentration was found to have a remarkable form of solutions: there is a tendency for establishing an intrinsic homogeneous concentration *c*_*hom*_ and at the apical and basal ends of the IC deviations from *c*_*hom*_ will occur. This behavior could be described by introducing the following novel parameters: a) the spatially homogeneous concentration of the osmolyte

$c_{hom}=\frac{c_{3}}{2}\left(1+\sqrt{1+\frac{4j}{f_{aq}c_{3}^{2}}}\right)$, which develops near the entrance part of the intercellular cleft. It depends on the cytosolic osmolyte concentration *c*_3_, on the ion flux due to ATPases *j* and on the transport coefficient of aquaporins *f*_*aq*_; b) the inhomogeneity length $y_{inh}=\sqrt{\frac{Dbc_{hom}}{j}}$ describes the characteristic distance from the TJ that is needed for *c*_*hom*_ to establish in the IC. This parameter depends on the diffusion coefficient of the osmolytes (*D*), on *c*_*hom*_ and, most important, on the width of the intercellular cleft *b*. Analytical approximations for the homogeneous regime and deviations from it near the apical and basal boundaries of the IC were derived showing that possible pathways of water and ion transport in epithelia are largely governed by these two parameters *c*_*hom*_ and *y*_*inh*_.

Our analysis predicts that the system can self-regulatory prevent water fluxes into the lumen through TJ and lateral membrane by varying the distance *b* between the epithelial cells. This mechanism is presumably very fast if there is a way in which the cells and/or the surrounding compartments modify the width *b*. Clearly the activity of LI-cadherins has the potential to modify the geometry, i.e. the width of the IC and thus to modify the water transport [12].

Our model suggests new possible scenarios of water flux regulation in response to osmolarity change in the lumen and consequently also in the cytosol. The cytosolic concentration growth *c*_3_ supports the water flux through TJ out of the lumen, and it can initiate the influx of water through lateral membrane into cytosol of the enterocyte. This would provide further epithelium absorption and decrease dangerous high osmolyte concentration in the cytosol (Figs 6 and 7). Our theoretical calculations are in agreement with experimental results [12]. They show that narrowing of the cleft provides: 1) change of water flux from negative to a positive, i.e. from into the lumen to out of the lumen even against the over all osmotic gradient (Figs 5C and 6C); 2) higher absorption rate, since a flux through the open end increases tremendously in narrow clefts (Fig 4C). As the cleft gets narrower, hyperosmolarity in the IC builds up, and water flux (velocity) rises. This agrees with faster hydration observed for fluids with higher concentration of electrolytes.

The functional role of LI-cadherins is yet debated. Their activation was recently linked to the concentration of Ca^2+^ (Fig 1B) [29], which can change via transcellular or paracellular pathways. Because Ca^2+^ ions obey the same laws as other osmolytes, our model also describes a potential scenario how LI cadherins facilitate water transport. In the case of low Ca^2+^-concentrations (~ low ion concentration *c*) LI-cadherin molecules are inactive and thus fail to keep the IC narrow. The binding of LI-cadherins in response to the increase in Ca^2+^ concentration can provide a fast mechanism for water flux regulation, suggesting that the IC acts as an osmotic sensor. We hypothesize, that as the osmolarity in the lumen elevates, LI-cadherin proteins can prevent water outflow to the lumen and facilitate the absorption.

This may happen as follows. Under hypoosmolarity conditions the LI-cadherins are deactivated (Fig 1B) and therefore the cleft is wide (*b*≈400*nm*). As hyperosmolarity in lumen builds up, the internal cell osmolarity rises (*c*_3_↑) and the spatially homogeneous concentration (*c*_*hom*_↑≈*c*(0)↑) increases as well (Eq 9 and Fig 7B).

As a result, LI-cadherins located in the region of higher concentration (Fig 7C), at the beginning of the cleft, become activated first, and narrow the cleft there (*b*≈40−100*nm*). This forces the system to squeeze water from a narrow region to a wider one due to additional hydrostatic pressure. Osmolyte diffusion creates high Ca^2+^ concentration near the basal end of IC. This positive feedback might activate LI-cadherin molecules further basal, thus squeezing water out of the channel. Fig 7 illustrates such a scenario.

Our theoretical finding shows that a simple epithelium as in the gut consisting mainly of enterocytes is a self-regulating system. This statement is due to the fact, that the enterocytes prevent both the outflow to the lumen and dangerous osmolarity in the cell by coupling the ion concentration at the cleft inlet, internal cell osmolarity and IC width. So high luminal osmolarities lead to high Ca^2+^-concentrations in the IC. This in turn narrows the IC or at least stabilizes a narrow IC. However, a narrow IC stabilizes the osmotic gradient from the lumen to the IC leading to a stable water flux and constant cytosolic osmolarity. This picture agrees with the steady state response of our model, to the variations of lumen and cell osmolarity and cleft width. Detailed analysis of this hypothesis requires consideration of temporal behavior with elastic equations for the lateral membrane in order to calculate the elastic deformations of the IC time dependent on the luminal conditions. This is beyond the scope of the current work, but shall be considered in future studies.

Our model suggests that the columnar epithelium is much more stable under hypertonic conditions than the simple cuboidal one. This can explain why such epithelia are found in organs with hypertonic conditions: airways (34), uterine tubes (35), the gall bladder [40,41] or small and large intestines. At the same time, the proximal tubule in the kidney, where reabsorption happens under isotonic conditions, has simple cuboidal epithelia.

LI-cadherin is found also in other organs with strong hypertonic conditions: gastrointestinal tract, the gall bladder and the appendix. This may help preventing water flux into the lumen of organs. Cadherin 16 with 7 extracellular domains is highly expressed in thyroid glands. Thyroid secretory epithelial cells enclose the colloid lumen, and their shape varies from flattened to columnar during hormone secretion. All this makes plausible, that LI-cadherin-like family contributes to the dynamic regulation of epithelium via changes in its geometry.

Thus, we suggested one of the possible regulating pathways, which adapts epithelia to changing environment within a broad osmotic range [42]. Paracellular transport constitutes about half of the water flux through the epithelium [22,43,44], and its geometry may play an important role among the mechanisms employed by various organs/organisms to maintain their physiological functions in different surroundings.

## Conclusions

The influences of geometry and physiological parameters onto the ion- and water-flux in simple epithelia were analyzed and discussed. Dimensionless analysis can be applied to a wide range of regimes in different transporting epithelia. We have shown that widening of the intercellular cleft (IC) can initiate flux into the lumen, explicitly through tight junctions or implicitly through the lateral membrane. Cleft narrowing under hyperosmolarity conditions alters the flux direction so that water flows out of the lumen. The behavior of the model agrees well with experimental data reported earlier (20), where the cleft width is regulated by LI-cadherins.

We suggest that LI-cadherin can greatly facilitate water absorption under hypertonic conditions by "automatically" narrowing the IC under hypertonic luminal conditions as a result of local elevation of Ca^2+^-levels. We further introduce the hypothesis that LI-cadherin molecules can be sequentially activated in a domino-like manner to squeeze water out of the intercellular cleft (Fig 7).

In general, our results estimate the ion- and water-uptake in a wide range of geometrical and physiological parameters. This helps to understand how epithelia function in various osmolarity contexts and can be the basis for further, more sophisticated models of epithelial transport.

## Supporting information

S1 Fig Influence of aquaporin density on ion concentration and water velocity in lateral membrane. (A) Concentration and (B) velocity profiles along the IC as aquaporin density changes. The following system parameters were used: *c*_3_ = 290*mM*, *c*_4_ = 300*mM*.
(TIF)

S2 Fig Influence of cytosolic osmolyte concentration *c*_3_ on the water flux distribution in the IC. (A) Water flows in the positive direction towards the interstitial tissue as long as *c*_3_<*c*(*y*)<*c*_*hom*_ in a long cleft, and changes its direction to negative when *c*_3_>*c*(*y*). It entails the inflow to the enterocyte from the IC. (B) In the case of a short cleft, a slight change in the cell concentration tends to change water flux direction to negative along the total IC as long as the condition *c*_3_>*c*(*y*) is fulfilled. The drawing is done based on numerical results presented in Fig 6.
(TIF)

S1 File Explanatory details. (DOCX)
