# Supplementary figures and images for: Physiological relevance of epithelial geometry: New insights into the standing gradient model and the role of LI cadherin
Source: PLoS One. 2018 Dec 21;13(12):e0208791. doi: 10.1371/journal.pone.0208791 (PMC6303100; doi:10.1371/journal.pone.0208791)

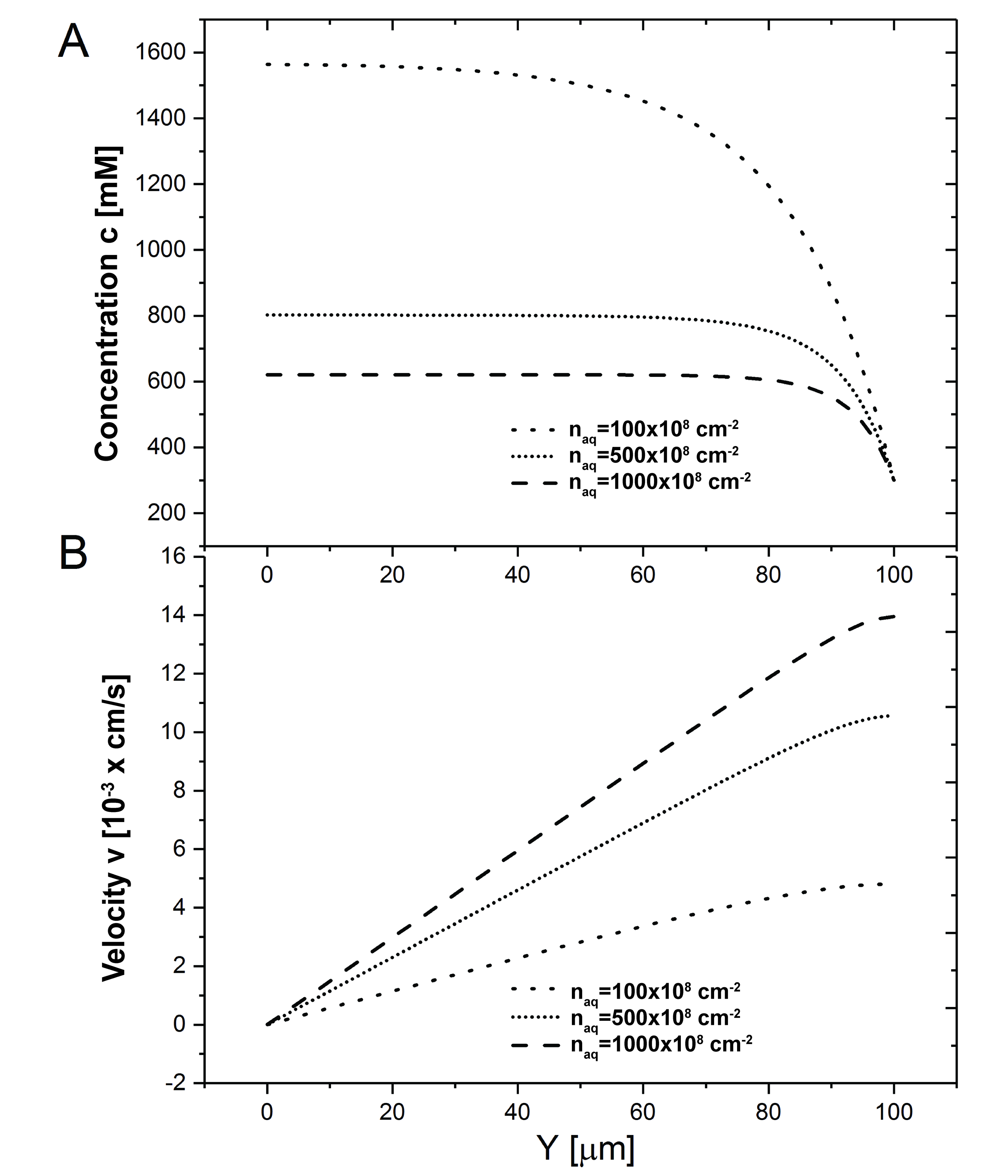

Supplement: S1 Fig — (A) Concentration and (B) velocity profiles along the IC as aquaporin density changes. The following system parameters were used: c3 = 290mM, c4 = 300mM. (TIF) [file pone.0208791.s001.tif]

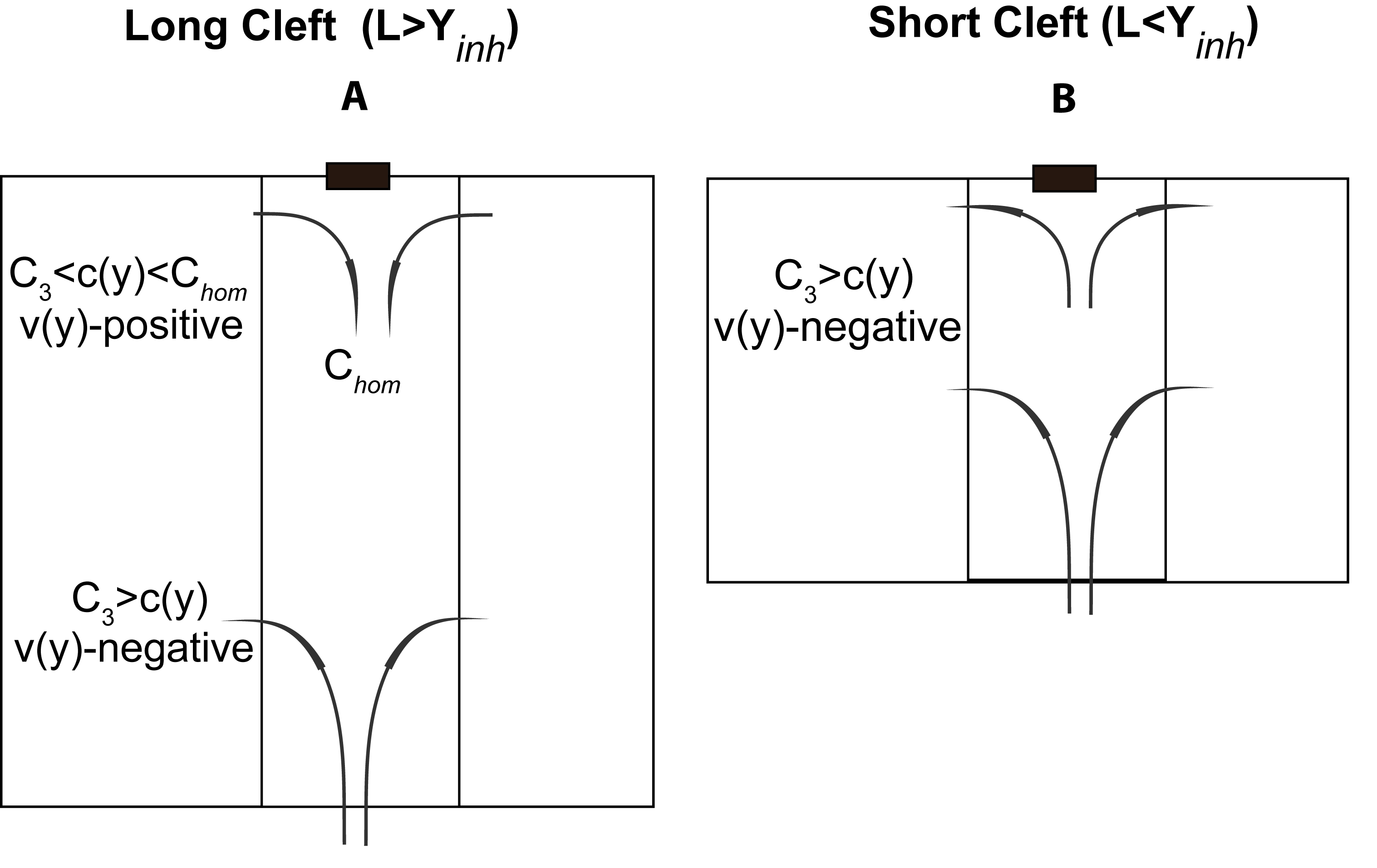

Supplement: S2 Fig — (A) Water flows in the positive direction towards the interstitial tissue as long as c3<c(y)c(y). It entails the inflow to the enterocyte from the IC. (B) In the case of a short cleft, a slight change in the cell concentration tends to change water flux direction to negative along the total IC as long as the condition c3>c(y) is fulfilled. The drawing is done based on numerical results presented in Fig 6. (TIF) [file pone.0208791.s002.tif]
