# Supplementary material for: Physiological relevance of epithelial geometry: New insights into the standing gradient model and the role of LI cadherin
Source: PLoS One. 2018 Dec 21;13(12):e0208791. doi: 10.1371/journal.pone.0208791 (PMC6303100; doi:10.1371/journal.pone.0208791)
Supplement: S1 File — (DOCX) [file pone.0208791.s003.docx]

**Supporting information**

**1. System of coupled stationary equations**

We start from the concentration, continuity and Navier-Stokes equations for incompressible water flow in 2D:

(17)

Here, $v^{y},\frac{\partial c}{\partial y}$ are the *y-the* component of the velocity and the derivative of the concentration with respect to *y*. Let us make two simplifications. First, we integrate all equations over *x* and use averaged quantities:

(18)

At this stage, we assume that $b=const$. Second, we assume that the *x*-velocity $v^{x}$ is given by the linear function, dictated by the boundary conditions, and the *y*-velocity $v^{y}$ follows a slowly varying quasi-Poiseuille flow. The validity of these assumptions can be tested and confirmed a posteriori.

(19)

Integration over *x* in (17) with the assumptions (19) and boundary conditions from (17) result in the following: the Navier-Stokes equation for $v^{x}$ is fulfilled automatically upon integration, as $0=0$ for symmetry reasons. This is even more general than the assumptions (19). Clearly, this does not hold for a strongly turbulent flow or (possibly) even for an asymmetric laminar flow at significant Reynolds numbers.

(20)

In the first equation, we have the expression $\overline{v^{y}c}$. Its calculation requires knowledge of the profile $c(x,y)$ along *x*. We assume that diffusion is so fast that any inhomogeneity of *c* *along* *x* is rapidly and almost completely smoothed, so we can write $\overline{v^{y}c}=\bar{v}^{y}\bar{c}$. Of course, due to the boundary conditions $(-D\frac{\partial c}{\partial y}+v^{x}c)\left|_{x=0,b}\right.=\pm j$, this is not exactly true, but since $D\Delta c/b\sim v^{x}c\sim v_{0}c$, the ratio $\Delta c/c\sim v_{0}b/D\ll1$ should be sufficiently small (Peclet number for the *x*-direction). Finally, considering the stationary case, using biological boundary conditions and substituting $\frac{\partial^{2}\bar{v}^{y}}{\partial y^{2}}$ in the last equation from the equations for $\frac{\partial\bar{v}^{y}}{\partial y}$, we obtain the equations with the boundary conditions in the following form for the quantities *averaged over x* (see definitions in ): $c\mathop{=}\limits^{def} \bar{c}, v\mathop{=}\limits^{def} \bar{v}^{y}, p\mathop{=}\limits^{def} \bar{p}$.

(21)

Hydrostatic pressure contributions to the aquaporin and TJ conductance are not included since they are small. Typical values are in the range $\Delta c=100mM$, and therefore $RT\Delta c=2.58\times10^{6}CGS$, while $\Delta p=0.1atm=10^{5}CGS$, which is about 26 times smaller. This is not the case if pressure influences the cleft width *b* via cleft elasticity, which requires an additional “elastic” equation. With these simplifications the reduced system of equations was obtained – see Eq. 1, 4 in the main text.

1. **Analytical approximations for long and short clefts**

It is convenient to combine both equations 11 into a single equation to obtain the deviation of the velocity profile $\delta v$ from its linear expression Eq. 10:

(22)

The first line represents a second-order non-linear ODE with respect to $\delta v$, while the second line can then be used to find $\delta c$. For further analysis, we introduce the dimensionless variables *V* and *Y*, defined as $\delta v=\bar{v}V, y=\bar{y}Y$, where $\bar{v}$ and $\bar{y}$ are initially unknown dimensional scales for velocity and length. We require that in dimensionless form the last two terms in Eq. 22 have the coefficient 1, which leads to the following definitions:

(23)

In dimensionless variables this yields:

(24)

Let us discuss the relative importance of these terms. Linear rescaling of *V* and *Y* does not change the relation between the first and the third terms on the right-hand side. Rescaling of *V* also does not influence them with respect to the left-hand side. Rescaling of *Y* can change the coefficient in to unity, keeping the ratio to *V*-term intact. Typically, $C_{4}\sim1$ and thus at the end of the cleft ($Y=L^{*}$) terms with *V* and $VV_{Y}$ are comparable, while at the beginning of the cleft ($Y=0$) the term *V* is inherently comparable to , as both are linear in *Y*:. Two situations can be distinguished: long and short clefts. The definition of “long” and “short” will be refined a posteriori based on their respective approximate solutions.

**Columnar epithelium: Long clefts with** $JL^{*}\gg1$ $(\mathrm{dimensional} \mathrm{form}:L\gg\frac{y_{inh}}{\sqrt{2}}\sqrt{\frac{f_{aq}c_{hom}^{2}}{j}})$

In long clefts the term is dominant near $Y=L^{*}$. This leads to the following solution:

(25)

The first set of equalities in Eq. 25 (b,c,d) refers to the situation in which the *V* term in the differential Eq. 25 a is retained, and is expressed in terms of the Kummer confluent hypergeometric function ${}_{1}F_{1}(a,b,z)$ (40). The terms $(Y/L^{*})^{1/J}$ should be retained, as it follows from Eq. 9 that $1/J>1$ always. For reference purposes we also provide the solutions to Eq. 25 a for non-zero velocity boundary condition $V(0)=V_{0}$, as defined by Eq. 11.

(26)

The second set of equalities in Eq. 25 (b,c,d) represents leading terms in .

The last line shows the relation between the different terms. The term dominates as long as $JL^{*}\gg1$, which defines where the first set of solutions is applicable. The second set of solutions also requires . This is a more restrictive condition for the length $L^{*}$ when $J\gg1$, and less restrictive when $J\ll1$.

**Cuboidal epithelium: short clefts with** $JL^{*}\ll1 (\mathrm{dimensional} \mathrm{form}: L\ll\frac{y_{inh}}{\sqrt{2}}\sqrt{\frac{f_{aq}c_{hom}^{2}}{j}})$ In short clefts, all terms in Eq. 24 are comparable, and no analytical solution can be found. The following approximation is suggested and supported by numerical studies. Clearly, in Eq. 24$C_{Y}(0)=0$ because $Y,V=0$ . Simultaneously, over the cleft length, *C* changes from (yet unknown) $C(0)=C_{0}$ to $C(L^{*})=C_{4}$, as dictated by the boundary conditions. The lowest order Taylor expansion suggests a parabolic profile for *C* and cubic behavior for *V*:

(27)

These expressions cannot satisfy the differential relation exactly, but they can fulfill it on average upon integration (method of moments (41). This yields a condition for $C_{0}$:

(28)

The last expression is a quadratic equation for $C_{0}$, which can be resolved as follows:

(29)

The second approximation is obtained from last of expressions Eq. 28, setting $C_{0}=C_{4}(1-\delta_{\ll1} )$ there. It is valid for short clefts with $\frac{L^{*^{2}}}{2}\left(J+1\right)\ll1\Rightarrow L^{2}\ll\frac{y_{inh}^{2}}{1+\frac{f_{aq}c_{hom}^{2}}{j}}$. When $C_{0}$ is found from Eq. 29, it is substituted into approximate *C-V* profiles 28. The simplest dimensional expressions based on expressions Eq. 25 (b,c) for long clefts, and equations 27, 29 for short ones, are discussed in the Result section (Eqs. 13 and 15).

**3. Hydrostatic Pressure**

The role of hydrostatic pressure was considered unimportant to system behavior. We discuss its influence hypothesizing that LI-cadherin can squeeze water from the IC. The following realistic boundary values were chosen: *p*_1_=0.03 atm, *p*_3_=0.03 atm, *p*_4_=0.01 atm.

**4. Calculation of the volume of water flux per day**

To assess the water flux through the TJ pores, we assume that each individual enterocyte has four shared attachments to other cells and recall that the calculated water flow through each attachment is shared by two cells. The number of TJs ($N_{TJ}$) per one square meter is therefore: $N_{TJ}=\frac{4\cdot surface\_area}{enterocyte\_width}\cdot\frac{1}{2}$. The volumetric flux for a day is calculated by: $Vol_{wf}=N_{TJ}\cdot24\cdot3600\cdot enterocyte\_width\cdot\bar{v}^{y}(y=0)\cdot b$, with $v\mathop{=}\limits^{def} \bar{v}^{y}$, for a cubic enterocyte with 20*μm* size and an IC width of $b=400$ $nm$.

**5. Comparison of numerical and analytical solutions**

The full nonlinear boundary problem Eq. 6 can be solved numerically using the “shooting” method (42): The boundary value problem is transformed into one of initial values that starts from $y=L$ and successively refines “shots” until a trajectory that has the desired value at $y=0$ is found. Success of a numerical algorithm therefore depends on the choice of initial values at $y=L$. Fast convergence is achieved if we estimate the velocity at $y=L$ by the homogeneous solution of Eq. 10 in dimensional form:

(30)

Figures 3 shows the numerical solutions for long and short clefts together with the approximations Eqs. 13 and 15. The exponential approximation Eq. 13 is valid for clefts $L>y_{inh}$, as confirmed numerically. The first parabolic approximation in Eq. 15 is applicable to short clefts with $L\ll\frac{y_{inh}}{\sqrt{2(1+\frac{f_{aq}c_{hom}^{2}}{j})}}$, which becomes $L<y_{inh}/2.4$ for typical values from Table 1. The second parabolic approximation in Eq. 15 helps to understand the qualitative influence of parameters on *y*-dependences.

**6. Influence of ATPases and aquaporins expression level on concentration**

ATPases density (Ion flux *j*) Clearly, larger ion flux increases cleft osmolarity, which enhances the water transfer out of the cleft. This is reflected in expression in Eq. 9 for a spatially homogeneous concentration $c_{hom}$.

Aquaporin density $n_{aq}$. Higher aquaporins density, or increase in their expression level, decreases the spatially homogeneous concentration, because $c_{hom}=\frac{c_{3}}{2}\left(1+\sqrt{1+\frac{4j}{f_{aq}c_{3}^{2}}}\right)$, where $f_{aq}=k_{aq}V_{wat}n_{aq}$. Simultaneously, homogeneous regime persists over a wider range $0<y<L-y_{inh}$ since decreases. One can see this effect in the S1 Figure. For example, for an aquaporin density of $n_{aq}=1000\cdot10^{8}cm^{-2}$, the concentration is approximately 620*mM* over the range [0;64] *μm*. For a ten times smaller aquaporin density $n_{aq}=100\cdot10^{8}cm^{-2}$, a very high value of concentration $c_{hom}$≈1550*mM* extends over a shorter cleft range [0;42] *μm* due to a smaller $y_{inh}$ value. Nevertheless, the flux out of the open end $v(L)b$ decreases, as aquaporins density grows (S1 Fig), since the velocity of water emerging through aquaporins is lower, because the osmotic difference between IC and internal cell concentration is lower.
